# Awareness and Support of Clinician- and Patient-Collected Human Papillomavirus Testing for Cervical Cancer Screening Among Primary Care Clinicians

**DOI:** 10.1089/whr.2021.0074

**Published:** 2022-01-07

**Authors:** Kathy L. MacLaughlin, Robert M. Jacobson, Jennifer L. St. Sauver, Gregory D. Jenkins, Chun Fan, Lila J. Finney Rutten

**Affiliations:** ^1^ Department of Family Medicine, Rochester, Minnesota, USA.; ^2^ Robert D. and Patricia E. Kern Center for the Science of Health Care Delivery, Rochester, Minnesota, USA.; ^3^ Division of Community Pediatric and Adolescent Medicine, Rochester, Minnesota, USA.; ^4^ Department of Quantitative Health Sciences, Rochester, Minnesota, USA.

**Keywords:** cervical cancer screening, primary HPV test

## Abstract

***Background:*** Cervical cancer screening has shifted toward human papillomavirus (HPV)-based testing, but uptake of primary HPV screening in the United States is unknown and previous studies highlight delays in clinician adoption of guideline updates.

***Methods:*** We conducted a cross-sectional electronic survey of primary care clinicians (*n* = 252; response rate = 30.9%) assessing awareness and support of primary HPV screening. We assessed factors for association with past use of HPV testing and support of clinician- and patient-collected HPV testing individually using Fisher's exact test and jointly using Firth's logistic regression.

***Results:*** Most clinicians (79%) were familiar with one or more primary HPV screening guidelines. Support for clinician-collected (89%) and patient-collected (82%) HPV testing was high, but only 34.5% reported prior use. Guideline familiarity was positively associated with HPV testing in practice (*p* = 0.0001). Support of clinician-collected testing was positively associated with more years in practice (*p* = 0.03), internal (vs. family) medicine specialty (*p* = 0.03), and guideline familiarity (*p* ≤ 0.0001). Male clinicians more frequently supported patient collection for patients overdue for screening (*p* = 0.013). Physicians more frequently than advanced practice providers (APPs) supported patient collection for screening-adherent women (*p* = 0.021). Multivariable analysis showed those unfamiliar with guidelines were less likely to have used HPV testing [odds ratio, OR: 0.10 (0.03–0.32)] or to support clinician-collected HPV testing [OR: 0.16 (0.07–0.37)]. APPs were less likely than physicians to support patient-collected HPV testing among screening-adherent women [OR: 0.42 (0.20–0.87)].

***Conclusions:*** We observed high levels of guideline awareness and clinician support for primary HPV testing, despite relatively low use. This merits further exploration to inform future interventions to increase uptake.

## Introduction

Cervical cancer is preventable through human papillomavirus (HPV) vaccination, screening, and treatment of precancers, yet estimates from the American Cancer Society (ACS) predict 14,480 cervical cancer cases and 4,290 deaths in the United States in 2021, occurring most often in never or underscreened women.^1^ Disparities in screening are also associated with sociodemographic factors, including race and ethnicity, household income, education, sexual orientation, and geography.^2^ Healthy People 2020 targeted increasing cervical cancer screening coverage to 93%.^2^ However, data from National Health Interview Survey (NHIS) reflect a concerning trend with a decline in screening rates from 2000 (86.5%) to 2018 (81.1%).^3,4^

Cervical cancer screening rates may be even lower as NHIS results are based on self-reported data, which have been shown to overestimate actual rates,^5^ as demonstrated by a report that only 64.6% of screening-eligible women were up to date with cervical cancer screening in 2016 using data from confirmed diagnosis and billing codes.^6^ Healthy People 2030 has since lowered the screening goal to 84.3%.^7^

With the recognition that persistent infection with high-risk strains of HPV (especially types 16 and 18) are associated with the development of cervical precancer and cancer, the paradigm for cervical cancer screening has shifted from a cytology-based (Pap) approach to an emphasis on HPV-based screening.^8^

In 2003, the Food and Drug Administration (FDA) approved the use of the combined Pap/HPV co-test, and in 2014, primary HPV testing was approved as a stand-alone option for cervical cancer screening.^9^ The following year, interim guidance was provided by the American Society of Colposcopy and Cervical Pathology (ASCCP) and Society of Gynecologic Oncology (SGO) supporting 3-year interval primary HPV testing as a cervical cancer screening option for women 25–65 years of age.^10^

The US Preventive Services Task Force (USPSTF) first endorsed the option of primary HPV screening in 2018 for women 30–65 years of age at 5-year intervals.^11^ The ACS released updated guidelines in 2020 recommending primary HPV testing as the preferred cervical cancer screening strategy for all women 25–65 years of age old with screening initiation at age 25.^12^

Uptake of primary HPV testing in the United States is unknown, but previous studies have highlighted delays among primary care providers in adopting cervical cancer screening guideline updates regarding screening frequency, test type, and patient age.^13–15^ Clinician perspectives related to use of primary HPV tests for screening published before the 2018 USPSTF update show knowledge gaps regarding efficacy of HPV testing and low levels of compliance with screening guidelines in terms of age of HPV test initiation and frequency.^16^

A novel application of primary HPV screening that addresses barriers to screening involves patient self-collection of a vaginal swab for HPV testing as high levels of concordance have been observed between patient-collected vaginal and clinician-collected cervical samples for HPV results^17^ and for detecting cervical precancers.^18^ Patient self-collection of a vaginal sample for HPV testing has been identified by the USPSTF as an innovative strategy that warrants ongoing research to evaluate impact on underscreened populations and to define the most successful implementation approaches.^11^

Adoption of new technologies such as primary HPV testing, per Diffusion of Innovations Theory, is impacted by attributes of the innovation, traits, and influencers of potential adopters, as well as the larger social context.^19^ We focused on assessing the potential adopters through a survey evaluation of primary care clinician perspectives on primary HPV screening. Our study addresses gaps in knowledge about clinician awareness, support, and use of primary HPV screening, following endorsement by the USPSTF, the principal authority for practice guidelines in primary care. In addition, we assess clinician support of screening through patient self-collection.

## Methods

We conducted an electronic cross-sectional survey of primary care clinicians and residents in Mayo Clinic practices to evaluate awareness and support of primary HPV testing for cervical cancer screening through clinician collection and patient self-collection, along with prior use of primary HPV testing in practice. The Institutional Review Board at Mayo Clinic approved this study.

### Survey design and content

Knowledge of and attitude toward an innovation are foundational steps in the adoption of new interventions per the innovation-decision stages outlined in the Diffusion of Innovations theory.^20^ These stages, which could also be described as awareness and support of an innovation, informed the focus of our survey design. Through a literature review, we identified survey domains, including clinician awareness and knowledge of primary HPV test guidelines, beliefs about the test, influencing factors on clinical practice, prioritized test attributes, and engagement with screening. These domains were used to develop survey items and provide content validity. We included several existing survey questions with the authors' permission as described below.

We developed investigator-generated questions for key survey question concepts not identified through literature review. We pretested a survey draft in the sampling frame population with probes to ensure questions were understandable and survey questions were measuring the intended concept to address construct validity.

Clinician demographics, including sex, race, and ethnicity, were collected along with medical practice characteristics of primary care specialty, degree, and years in practice. To assess awareness of the USPSTF and ACS guidelines, a paragraph was embedded in the survey describing primary HPV testing and explaining current USPSTF and ACS guidelines related to primary HPV screening. Survey respondents were then asked if they were aware of USPSTF, ACS, both, or neither guideline. Clinician use of primary HPV testing in practice was assessed by self-report.

Clinicians who reported not using primary HPV testing for cervical cancer screening were asked to select one main reason from the following response options: (1) I am not comfortable with the primary HPV testing option at this time; (2) My patients would not be comfortable with primary HPV testing; (3) It is not standard practice at my clinic; or (4) Other, please specify (open-ended space provided). We also included with author permission a question from a 2015 survey of US providers asking to what extent cervical cancer screening practices were influenced by the following factors: practice guidelines, clinical experience, patient preference, and patient HPV vaccination status.^21^

We asked clinicians if they were familiar with the concept of patient self-collection of a vaginal sample for HPV testing, for cervical cancer screening. Questions modified with permission from Mao et al.^22^ queried respondents about attributes of patient-collected vaginal HPV tests they considered most important, support for and concerns with this approach. Clinicians answered if they strongly supported, somewhat supported, somewhat opposed, or strongly opposed the three following scenarios for cervical cancer screening: (1) clinician-collected primary HPV test as supported by USPSTF and ACS guidelines; (2) patient-collected primary HPV test for women overdue or never screened; and (3) patient-collected primary HPV test for women historically adherent to screening.

Clinicians were advised to answer as if there was FDA approval for the patient collection scenarios. Currently, there are no FDA-approved HPV self-sampling kits. There are several companies in the United States that offer HPV self-sampling kits for patients, but these are unregulated and not recommended by the national cervical cancer screening guideline groups. For analysis, we dichotomized those results into “support” (strongly or somewhat) or “oppose” (somewhat or strongly).

### Population

The sampling frame included all Family Medicine (FM) and Internal Medicine (IM) physicians and nurse practitioner and physician assistant providers grouped together as advanced practice providers (APP), as well as FM residents in the Mayo Clinic Primary Care practices. Administrators in coverage regions confirmed 815 clinicians/residents in the sampling frame. We obtained permission to conduct the survey from department and division chairs of FM and IM and Mayo Clinic Human Resources. As the primary outcome variables were single proportion estimates, we based the desired sample size on calculations of precision.

For a 95% confidence level that the true primary outcome values were within a 5% margin of error of the reported values and using the population size of 815 and 50% population proportion, the desired sample size was calculated to be 262. We invited all identified clinicians to participate in the survey as a census study sample to target the desired sample size. Clinicians who reported not performing cervical cancer screening in their clinical practice (first question of the survey) were subsequently excluded.

### Survey administration and data collection

The Mayo Clinic Survey Research Center performed the survey administration and data collection. As per the Dillman method,^23^ a multiple contact data collection protocol, including up to three contacts, was deployed. We sent a Qualtrics unique web survey link to the clinicians' Mayo Clinic work e-mail addresses. Two weeks later, we sent a follow-up email with a link to the web survey to nonresponders. After another 2 weeks, all remaining nonrespondents received a third and final email survey invitation and link.

### Outcomes

Primary outcome variables included the following: (1) prior use of primary HPV testing in clinical practice; (2) support for clinician-collected primary HPV screening; (3) support for patient-collected primary HPV screening in underscreened women; and (4) support for patient-collected primary HPV screening in women historically adherent to screening (defined as Pap test in the last 3 years, Pap/HPV co-test in the last 5 years, or primary HPV test in the last 5 years).

Secondary outcomes included prior use of primary HPV testing in practice and support of primary HPV testing in the three different scenarios by clinician demographics of sex, race/ethnicity, type of clinician, years in practice, clinical specialty, factors influencing cervical cancer screening practices, and awareness of guidelines.

### Statistical analyses

Descriptive statistics were reported for clinician demographics, practice characteristics, factors influencing cervical cancer screening practices, and USPSTF and ACS guideline awareness. In the descriptive table, we reported rates of guideline awareness by categories of USPSTF, ACS, both, or neither.

In the bivariate and multivariate analyses, we grouped awareness as “yes” (aware of one or both guidelines) or “no” (aware of neither guideline). Bivariate associations between clinician characteristics and prior use of primary HPV testing in practice, and support for primary HPV testing by clinician collection and by patient self-collection in underscreened compared with screening-adherent women were tested using Fisher's exact tests due to the rarity of survey responses for some responses. We calculated confidence intervals for odds ratios (ORs) of single predictors by inverting Fisher's exact tests.

To assess the simultaneous association between multiple predictors and the previously described outcomes, we first considered the correlation of predictors and selected predictors that were better targets for future intervention. Tests for pairwise association of all predictors were conducted and predictors in multivariable models were included for their actionable and practice relevance.

For example, type of clinician was highly correlated with sex of the clinician. Future educational interventions would more appropriately be targeted based on clinician type (physician/resident vs APP) rather than by clinician sex, so sex was dropped as a predictor variable. In addition, a high correlation was observed among type of clinician, years of experience, and clinical specialty; targeting an intervention to the type of clinician would be more feasible than targeting years of experience.

For the multivariate models, we only included variables that were significant for at least one of the outcomes in the bivariate analysis: clinician type, influence of practice guidelines, and guideline familiarity. We used Firth's logistic regression to model the outcomes with the selected predictors due to rarity of one of the categories of the outcomes; also, Firth's logistic regression, being a penalized regression model, is more stable in the presence of correlated predictors.

## Results

### Demographic characteristics of study population

Table 1 summarizes descriptive demographic and practice characteristics of the study population overall and by clinician type. We sent the survey to 815 Mayo Clinic primary care clinicians and 252 responded (30.9% response, margin of error 5.13%), with 16 clinicians reporting that cervical cancer screening was not part of their clinical practice. Those 16 clinicians were not included in further analysis (*n* = 236 for final analysis; margin of error 5.38%). Most survey respondents were female (73.2%), white (90.7%), non-Hispanic (97.0%), in practice <20 years (69.0%), FM clinicians (86.0%), and practicing in the Midwest (88.1%).

**Table 1.** Demographic and Practice Characteristics of Clinicians (*n*, %)

	Physician and resident (*n* = 144)	APP^a^ (*n* = 92)	Total (*n* = 236)
Sex			
Male	60, 42.0%	3,3.3%	63,26.8%
Female	83, 58.0%	89,96.7%	172,73.2%
Race			
White	125, 86.8%	89, 96.7%	214, 90.7%
Black	2, 1.4%	1, 1.1%	3, 1.3%
Asian	13, 9.0%	0	13, 5.5%
Other	1, 0.7%	0	1, 0.4%
Unknown	3, 2.1%	2, 2.1%	5, 2.1%
Ethnicity			
Hispanic	4, 2.8%	1, 1.1%	5, 2.1%
Not Hispanic	139, 96.5%	90, 97.8%	229, 97.0%
Unknown	1, 0.7%	1, 1.1%	2, 0.9%
Years in practice			
<10	46, 31.9%	57, 62.0%	103, 43.6%
10–19	42, 29.2%	18,19.6%	60, 25.4%
20–29	33, 22.9%	16, 17.4%	49, 20.8%
>30	23, 16.0%	1, 1.1%	24, 10.2%
Clinical specialty			
FM	119, 82.6%	84, 91.3%	203, 86.0%
IM	25, 17.4%	8, 8.7%	33, 14.0%
Practice region			
Midwest	125, 86.8%	83, 90.2%	208, 88.1%
Others	19, 13.2%	9, 9.8%	28, 11.9%
Factors influencing cervical cancer screening practices			
Practice guidelines			
Not at all	0	0	0
Slightly	1, 0.7%	0	1, 0.4%
Somewhat	5, 3.5%	1, 1.1%	6, 2.6%
Very much	137, 95.8%	91, 98.9%	228, 97.0%
Clinical experience			
Not at all	14, 9.9%	14, 15.2%	28, 12.0%
Slightly	26, 18.3%	9, 9.8%	35, 15.0%
Somewhat	58, 40.9%	26, 28.3%	84, 35.9%
Very much	44, 31.0%	43, 46.7%	87, 37.1%
Patient preference			
Not at all	10, 7.0%	6, 6.6%	16, 6.9%
Slightly	43, 30.3%	31, 34.1%	74, 31.8%
Somewhat	60, 42.3%	30, 33.0%	90, 38.6%
Very much	29, 20.4%	24, 26.4%	53, 22.8%
Patient HPV vaccine status			
Not at all	81, 57.5%	53, 57.6%	134, 57.5%
Slightly	15, 10.6%	11, 12.0%	26, 11.2%
Somewhat	21, 14.9%	12, 13.0%	33, 14.2%
Very much	24, 17.0%	16, 17.4%	40, 17.2%
Awareness of screening recommendations^b^			
USPSTF	54, 37.5%	28, 30.4%	82, 34.8%
ACS	0, 0%	6, 6.5%	6, 2.5%
Both	63, 43.8%	35, 38.0%	98, 41.5%
Neither	27, 18.8%	23, 25.0%	50, 21.2%

Missing survey responses not included in table.

^a^ Nurse practitioner or physician assistant.

^b^ Cervical cancer screening recommendations from USPSTF (2018) and ACS (2020).

ACS, American Cancer Society; APP, advanced practice provider; FM, Family Medicine; HPV, human papillomavirus; IM, Internal Medicine; USPSTF, US Preventive Services Task Force.

Clinician ranking of factors that influence their cervical cancer screening practices “very much” (with other options of “not at all,” “slightly,” or “somewhat”) included practice guidelines (97.0%), clinical experience (37.1%), patient preference (22.8%), and patient HPV vaccine status (17.2%). Among those surveyed, 34.8% were aware of the USPSTF guidelines, 2.5% with ACS guidelines only, 41.5% with both guidelines, and 21.2% were not familiar with either guideline (Table 1).

Among the survey respondents, 34.5% reported prior use of primary HPV testing in their clinical practice. The foremost reason for nonuse among the 65.5% of clinicians with no prior use of primary HPV testing was the perception that it is not standard practice at their clinic (68.9%). Only 9.3% reported personal discomfort with the test option and 1.3% expressed that their patients would not be comfortable with the option. Among the 20.5% who selected the option of “other” with a free text box available, we grouped the answers into identified themes that included lack of awareness of national guidelines, concern about evidence supporting primary HPV testing, and not knowing how to order the test in the electronic medical record.

Clinician awareness of the concept of patient self-collection of a vaginal sample for HPV testing was reported by 42.8% of survey respondents.

When asked to select the three most important test characteristics for patient self-collection, clinicians selected patient ability to obtain an adequate sample (87.7%), test sensitivity (82.6%), and patient acceptability (47.5%). The most common concern about patient self-collection for HPV testing was the missed opportunity to address other health issues (64.3%) followed by concern that women would not return for other care (11.5%). A small number of clinicians were of the opinion that only the provider should perform cervical cancer screening (6.4%) and 17.8% of survey respondents expressed no concern about patient self-collection.

### Association of clinician characteristics with prior use of and support for primary HPV testing

Results of bivariate analysis for associations between clinician characteristics and prior use of primary HPV testing, and support for clinician and patient-collected primary HPV testing for cervical cancer are presented in Table 2. Clinician characteristics associated with prior use of primary HPV testing included reporting practice guidelines as a strong influence on clinic practice (*p* = 0.048) and familiarity with guidelines (*p* = 0.0001). We observed a high level of support for the three screening scenarios presented: 88.9% of survey respondents were supportive of clinician-collected primary HPV testing; 81.8% were supportive of patient-collected primary HPV testing for women overdue or not previously screened; and 86.0% were supportive of patient-collected primary HPV testing for women previously adherent to screening.

**Table 2.** Association Between Clinician Characteristics and Prior Use of Primary Human Papillomavirus Testing in Practice and Support of Clinician- or Patient-Collected Primary Human Papillomavirus Testing for Cervical Cancer Screening

	Prior use of primary HPV testing in practice	*p*	Support^a^ clinician-collected primary HPV test	*p*	Support^a^ patient-collected primary HPV test (screening overdue or never done)	*p*	Support^a^ patient-collected primary HPV test (past adherence to screening)	*p*
Total	81, 34.5%		209, 88.9%		193, 81.8%		202, 86.0%	
Sex		0.2136		0.4826		0.0129		0.5277
Male	26, 41.3%		58, 92.1%		58, 92.1%		56, 88.9%	
Female	54, 31.6%		150, 87.7%		134, 77.9%		145, 84.8%	
Race and Ethnicity		0.1330		0.7476		0.4298		0.7762
White and (non-Hispanic or unknown ethnicity)	68, 32.7%		184, 88.5%		169, 80.9%		178, 85.6%	
Others	13, 48.2%		25, 92.6%		24, 88.9%		24, 88.9%	
Type of clinician		0.7785		0.1353		0.3008		0.0207
Physician and resident	51, 35.4%		131, 91.6%		121, 84.0%		130, 90.3%	
APP (NP and PA)	30, 33.0%		78, 84.8%		72, 78.3%		72, 79.1%	
Years in practice		0.6812		0.0301		0.2837		0.5357
<10	36, 35.3%		85, 83.3%		81, 78.6%		89, 87.3%	
10–19	18, 30.0%		54, 90.0%		48, 80.0%		49, 81.7%	
20+	27, 37.0%		70, 95.9%		64, 87.7%		64, 87.7%	
Clinical specialty		0.5560		0.0306		0.0535		1.000
FM	68, 84.0%		177, 87.2%		162, 79.8%		173, 85.6%	
IM	13, 16.1%		32, 100%		31, 93.9%		29, 87.9%	
Factors strongly influencing cervical cancer screening practices^b^								
Practice guidelines		0.0478		0.5665		0.3574		0.5974
Yes	75, 33.0%		202, 89.0%		186, 81.6%		194, 85.5%	
No	5, 71.4%		6, 85.7%		7, 100%		7, 100%	
Clinical experience		0.3210		0.5274		0.1580		0.3307
Yes	33, 37.9%		78, 90.7%		67, 77.0%		71, 82.6%	
No	46, 31.5%		129, 87.8%		125, 85.0%		129, 87.8%	
Patient preference		0.3283		0.4592		0.8408		0.6548
Yes	21, 39.6%		49, 92.5%		43, 81.1%		46, 88.5%	
No	58, 32.4%		157, 87.7%		148, 82.2%		153, 85.0%	
Patient HPV vaccine status		0.8533		0.5839		0.2569		0.6153
Yes	14, 35.0%		37, 92.5%		30, 75.0%		36, 90.0%	
No	64, 33.3%		169, 88.0%		161, 83.4%		164, 85.4%	
Awareness of screening recommendations^c^		0.0001		<0.0001		0.8366		0.4918
Yes	78, 42.2%		174, 94.1%		151, 81.2%		157, 84.9%	
No	3, 6.0%		35, 70.0%		42, 84.0%		45, 90.0%	

Missing survey responses not included in table.

^a^ Reported as “somewhat support” or “strongly support.”

^b^ Reported as “very much” vs. all others.

^c^ Cervical cancer screening recommendations from USPSTF (2018) and ACS (2020).

NP, nurse practitioner; PA, physician assistant.

However, specific clinician characteristics were associated with a greater likelihood of support for clinician-collected primary HPV testing. An increase in practice years was associated with higher levels of support (*p* = 0.03). IM clinicians were universally supportive (100.0%) compared with FM clinicians (87.2%; *p* = 0.03). Higher levels of support for clinician-collected primary HPV testing were also observed among those who were aware of the USPSTF and/or ACS guidelines (94.1%) compared to those who were not familiar with the guidelines (70.0%; *p* < 0.0001).

Clinician support of patient-collected primary HPV testing in women never screened or overdue for screening was significantly higher among male clinicians (92.1%) compared with female clinicians (77.9%; *p* = 0.013). No significant differences were observed by other clinician characteristics. Clinician support of patient-collected primary HPV testing in women historically adherent to screening was only associated with type of clinician, with higher support observed among physicians/residents (90.3%) than APPs (79.1%; *p* = 0.021).

### Significant predictors of clinician prior use of and support for primary HPV testing

We assessed the joint association of clinician characteristic predictors with the following outcomes: (1) prior use of primary HPV testing in practice, (2) support for clinician-collected primary HPV testing, (3) support for patient-collected primary HPV testing for women never screened or underscreened, and (4) support for patient-collected primary HPV testing for women historically adherent to screening. Our multivariable models included clinician type, influence of practice guidelines, and guideline familiarity as predictors (Table 3).

**Table 3.** Joint Association of Clinician Characteristics with Prior Use of Primary Human Papillomavirus Testing in Practice and Support of Clinician- or Patient-Collected Primary Human Papillomavirus Testing for Cervical Cancer Screening

Outcome	Predictors	Unadjusted		Adjusted	
		OR (95% CI)	*p*	OR (95% CI)	*p*
Prior use of primary HPV testing in practice	Type of clinician		0.7785		0.8266
	Physician and resident	Ref.		Ref.	
	NP and PA	0.90 (0.49–1.62)		1.07 (0.59–1.93)	
	Practice guidelines influence^b^		0.0478		0.0847
	Yes	Ref.		Ref.	
	No	0.20 (0.02–1.25)		0.21 (0.04–1.24)	
	Awareness of screening recommendations^c^		0.0001		<0.0001
	Yes	Ref		Ref	
	No	0.09 (0.02–0.29)		0.10 (0.03–0.32)	
Support^a^ clinician-collected primary HPV test	Type of clinician		0.1353		0.1703
	Physician and resident	Ref.		Ref.	
	NP and PA	0.51 (0.20–1.26)		0.55 (0.23–1.29)	
	Practice guidelines influence^b^		0.5665		0.3438
	Yes	Ref.		Ref.	
	No	1.35 (0.03–11.81)		2.74 (0.34–22.16)	
	Awareness of screening recommendations^c^		<0.0001		<0.0001
	Yes	Ref.		Ref.	
	No	0.15 (0.06–0.38)		0.16 (0.07–0.37)	
Support patient-collected primary HPV test (screening overdue or never done)	Type of clinician		0.3008		0.2434
	Physician and resident	Ref.		Ref.	
	NP and PA	0.68 (0.33–1.42)		0.67 (0.34–1.31)	
	Practice guidelines influence^b^		0.3574		0.4646
	Yes	Ref.		Ref.	
	No	0 (0–2.45)		0.32 (0.02–6.86)	
	Awareness of screening recommendations^c^		0.8366		0.6676
	Yes	Ref.		Ref.	
	No	1.22 (0.50–3.27)		1.20 (0.52–2.76)	
Support patient-collected primary HPV test (past adherence to screening)	Type of clinician		0.0207		0.0204
	Physician and resident	Ref.		Ref.	
	NP and PA	0.41 (0.18–0.92)		0.42 (0.20–0.87)	
	Practice guidelines influence^b^		0.5974		0.6205
	Yes	Ref.		Ref.	
	No	0 (0–3.28)		0.46 (0.02–10.24)	
	Awareness of screening recommendations^c^		0.4918		0.3018
	Yes	Ref.		Ref.	
	No	1.61 (0.57–5.62)		1.68 (0.63–4.51)	

Missing survey responses not included in table.

^a^ Reported as “somewhat support” or “strongly support.”

^b^ Reported as “very much” vs. all others.

^c^ Cervical cancer screening recommendations from USPSTF (2018) and ACS (2020).

CI, confidence interval; OR, odds ratio.

Compared with clinicians familiar with the USPSTF and ACS cervical cancer screening guidelines (referent), those unfamiliar with the guidelines were significantly less likely to report prior use of primary HPV testing in their practices [adjusted OR 0.10 (0.03–0.32)] and significantly less likely to support clinician-collected primary HPV testing for cervical cancer screening [adjusted OR 0.16 (0.07–0.37)].

There were no significant predictors identified for clinician support of patient-collected primary HPV testing for cervical cancer screening among women overdue for screening or never screened. APP primary care providers were less likely to support patient-collected primary HPV testing for cervical cancer screening among women with past adherence to screening [adjusted OR 0.42 (0.20,0.87)] compared with physicians and residents (referent).

## Discussion

Among clinicians responding to our survey study, use of primary HPV testing in clinical practice was low, despite high levels of awareness and reported influence of relevant national guidelines. Our results were generally consistent with results of a survey of physicians conducted 3 months after the ASCCP/SGO 2015 interim guidance publication on primary HPV testing where 40.8% of physicians reported routine use of primary HPV screening.^21^ Our results also revealed high levels of support for both clinician-collected and patient-collected HPV testing. The observed discordance between prior use of primary HPV testing in practice compared with reported guideline awareness and support illustrates the challenge of implementing practice change in real-world clinical settings.

In 2020, primary HPV screening was included as an acceptable performance measure of cervical cancer screening in the Healthcare Effectiveness Data and Information Set (HEDIS^®^); the addition of this metric may improve interest in and uptake of primary HPV testing in clinical practice as an HPV test alone will “count” toward health care systems quality metrics.^24^

The most frequently reported reason for no prior use of primary HPV testing among the clinician survey respondents in our study was their perception that it is not standard practice at their clinic. Other identified barriers included not realizing the test was available or how to order it, lack of awareness of national guidelines or of the evidence supporting primary HPV testing, as well as provider and perceived patient discomfort with the test option.

Educational interventions could be developed to target these specific barriers, although normalizing primary HPV screening as a standard practice option would likely take greater effort. A recent qualitative study from the Kaiser Permanente Southern California health system noted similar clinician concerns about both the evidence for transitioning to primary HPV testing and the test option not being standard practice in other community clinics.^25^

Additional barriers that were not identified in our study included concerns about increased provider workload associated with anticipated patient questions about primary HPV testing and the need to learn a new algorithm to manage abnormal results. Proposed facilitators to implementing a change to primary HPV screening included development of a brief evidence-based primary HPV test fact sheet and a management flowsheet both directed to clinicians and providing a script for clinicians to address patient concerns.^25^

Beyond clinician support for guideline-based clinician-collected primary HPV tests for screening, understanding clinician support for patient collection as an alternative screening option to address screening disparities will become increasingly important once the FDA approves a patient HPV self-test kit. Efforts are currently underway, with support from the National Cancer Institute's Division of Cancer Prevention, to validate patient self-collected HPV testing as a “noninferior” option to clinician collection and to “formally lay out a pathway for regulatory approval for self-sampling approaches for HPV testing” through the “Last Mile Initiative: Self-sampling for HPV testing to Improve Cervical Cancer Prevention” Trial.^26^

In our study, clinician support of patient self-collection of a vaginal HPV swab for primary HPV testing was comparable to support reported for clinician-collected primary HPV testing. The main concern among clinicians in our survey with patient self-collection was the potential for a missed opportunity to address other health issues, which is consistent with previously published research.

In a qualitative study exploring Canadian stakeholder attitudes toward patient self-collection, clinician concern about lost opportunity to address other health issues was a common theme.^27^ Missed opportunity was reported as a reason to not recommend patient self-collection in a clinician survey at the University of Washington as well, although acceptability of patient self-collection for HPV testing was reported if certain test characteristics were met, including high sensitivity, cost effectiveness, patient acceptability, and ability of patient to obtain an adequate sample, similar to reasons endorsed by clinicians in our survey.^22^

### Strengths and limitations

Strengths of our study include providing updated data on clinician-reported support for and use of primary HPV testing following endorsement by USPSTF in 2018 and following ACS's recommendation of primary HPV testing as the preferred cervical cancer screening option in 2020. We also identified barriers to primary HPV testing in clinical practice. This information may be used to inform future interventional studies to promote uptake of this preferred screening method. In addition, we provided an update to the knowledge base about support among primary care clinicians for a patient self-collection option, for primary HPV testing.

As an inherent limitation of a cross-sectional design, causality cannot be determined by our study results. The survey respondents were primarily white, non-Hispanic, female, FM clinicians in the Midwest and as such, results may not be representative of a different population of clinicians. The response rate was relatively low at 30.9%. In addition, the survey data on primary HPV testing use in clinical practice were based on clinician self-report and not confirmed with patient chart reviews or billing data, which would likely improve accuracy.

## Conclusions

Despite high levels of clinician awareness and support of guidelines, reported as strongly influential on clinical practice, we observed relatively low use of primary HPV testing as a cervical cancer screening option in the surveyed population. Educational interventions for clinicians should be developed to target identified barriers to the uptake of this evidence-based screening test option in clinical practice.

## Abbreviations Used

ACS: American Cancer Society
APP: advanced practice provider
ASCCP: American Society of Colposcopy and Cervical Pathology
CI: confidence interval
FM: Family Medicine
FDA: Food and Drug Administration
HPV: human papillomavirus
IM: Internal Medicine
NHIS: National Health Interview Survey
NP: nurse practitioner
OR: odds ratio
PA: physician assistant
SGO: Society of Gynecologic Oncology
USPSTF: US Preventive Services Task Force
